# Supplementary material for: Statistical ensembles without typicality
Source: Nat Commun. 2018 Mar 9;9:1022. doi: 10.1038/s41467-018-03230-y (PMC5845005; doi:10.1038/s41467-018-03230-y)
Supplement: Supplementary file 1 — Supplementary Information [file 41467_2018_3230_MOESM1_ESM.pdf]

# Supplementary Material - Statistical ensembles without typicality

Boes et al.

## SUPPLEMENTARY METHODS 1
## PROOF OF OF MAIN RESULT

### General maximum entropy ensembles

In this section we generalize the formalism laid out in the results section of the main text to the case of many conserved quantities. That is, the macrostate and microstate operations and the notion of operational equivalence are generalised to the more general case of a set $\{Q^j\}$ of $n$ commuting observables replacing $H$, and a set $\{v^j\}$ of expectation values for each observable replacing $e$. We introduce the following notation to arrange these sets into vectors

$$\mathcal{Q} = (Q^1, \ldots, Q^n), \tag{1}$$
$$\mathbf{v} = (v^1, \ldots, v^n), \tag{2}$$

so the macrostate of the system is given by $(\mathbf{v}, \mathcal{Q})$. The equivalence class of quantum states compatible with the macrostate is denoted by $[\mathbf{v}]_{\mathcal{Q}}$.

We model the environment with an analogous assumption as i) in the main text, but for the case of more conserved quantities. We assume that one can have access to $N$ uncorrelated subsystems described each by a macrostate. The mean value of the conserved quantities is determined by the value of a vector of inverse "temperatures" $\boldsymbol{\beta} = (\beta_1, \ldots, \beta_n)$ for each conserved quantity. We denote, say for subsystem $E^l$, the conserved quantities and mean values as

$$\mathcal{Q}_{\mathrm{E}^l} = (Q^1_{\mathrm{E}^l}, \ldots, Q^j_{\mathrm{E}^l}), \tag{3}$$
$$\mathbf{v}_{\boldsymbol{\beta}}(\mathcal{Q}_{\mathrm{E}^l}) = \big(v_{\boldsymbol{\beta}}(Q^1_{\mathrm{E}^l}), \ldots, v_{\boldsymbol{\beta}}(Q^j_{\mathrm{E}^l})\big), \tag{4}$$

where we are making the slight abuse of notation to identify

$$Q^1_{\mathrm{E}^l} := \mathbb{I}_1 \otimes \cdots \otimes Q^1_{\mathrm{E}^l} \otimes \cdots \otimes \mathbb{I}_N. \tag{5}$$

In this way, we will denote the $j$-th conserved quantity on the whole environment as $Q^j_{\mathrm{E}} = \sum_{l=1}^N Q^j_{\mathrm{E}^l}$. Note that $Q^j_{\mathrm{E}}$ plays a similar role as the Hamiltonian of the environment $H_{\mathrm{E}}$ in the main text, but in this case for a different conserved quantity. Accordingly we also can arrange the conserved quantities of the environment, and the compound SE in a vector as

$$\mathcal{Q}_{\mathrm{E}} = (Q^1_{\mathrm{E}}, \ldots, Q^n_{\mathrm{E}}), \tag{6}$$
$$\mathcal{Q}_{\mathrm{SE}} = (Q^1 + Q^1_{\mathrm{E}}, \ldots, Q^n + Q^n_{\mathrm{E}}). \tag{7}$$

The environment is modeled by any macrostate of the form $\bigotimes_{l=1}^N (\mathbf{v}_{\boldsymbol{\beta}}(\mathcal{Q}_{\mathrm{E}^l}), \mathcal{Q}_{\mathrm{E}^l})$ where, in analogy to equation $(4)$ of the main text, we assign a mean value of the conserved quantities equal to the "thermal" value, which in this case corresponds to the value that a maximum-entropy ensemble takes. That is,

$$v_{\boldsymbol{\beta}}(Q^j_{\mathrm{E}^l}) = \mathrm{tr}\left(\gamma_{\boldsymbol{\beta}}(\mathcal{Q}_{\mathrm{E}^l})\, Q^j_{\mathrm{E}^l}\right), \tag{8}$$

where $\gamma_{\boldsymbol{\beta}}$ is the so-called generalised Gibbs ensemble (GGE) defined as

$$\gamma_{\boldsymbol{\beta}}(\mathcal{Q}_{\mathrm{E}^l}) := \frac{e^{-\sum_j \beta_j Q^j_{\mathrm{E}^l}}}{\mathrm{tr}\left(e^{-\sum_j \beta_j Q^j_{\mathrm{E}^l}}\right)}. \tag{9}$$

We are now in a position to introduce macrostate operations.

*Definition* 1 (Macrostate operations with many charges) We say that $\rho_{\mathrm{f}}$ can be reached by macrostate operations from $(\mathbf{v}, \mathcal{Q})$, which we denote by

$$(\mathbf{v}, \mathcal{Q}) \overset{\boldsymbol{\beta}\text{-mac}}{\rightarrow} \rho_{\mathrm{f}}, \tag{10}$$

if for any $\epsilon > 0$ and $\epsilon' > 0$ there exist an environment with observables $\mathcal{Q}_{\mathrm{E}}$, and a unitary on SE such that

$$\left\|\mathrm{tr}_{\mathrm{E}}(U(\rho_{\mathrm{i}} \otimes \rho_{\mathrm{E}^1} \otimes \cdots \otimes \rho_{\mathrm{E}^m})U^\dagger) - \rho_{\mathrm{f}}\right\|_1 \leq \epsilon, \tag{11}$$

while preserving the global value of all the charges

$$\left|\mathrm{tr}\left(U(\rho_{\mathrm{i}} \bigotimes_{l=1}^N \rho_{\mathrm{E}^l})U^\dagger\, Q^j_{\mathrm{SE}}\right) - \mathrm{tr}\left(\rho_{\mathrm{i}} \bigotimes_{l=1}^N \rho_{\mathrm{E}^l}\, Q^j_{\mathrm{SE}}\right)\right| \leq \epsilon', \tag{12}$$

for all $j = 1, \ldots, n$. Importantly, both Supplementary Equations (11) and (12) have to be fulfilled for all the states of S and E compatible with our partial information, that is,

$$\forall \rho_{\rm i} \in [\mathbf{v}]_{\mathcal{Q}},\ \rho_{{\rm E}^l} \in [\mathbf{v}_{\boldsymbol{\beta}}(\mathcal{Q}_{{\rm E}^l})]_{\mathcal{Q}_{{\rm E}^l}} \text{ for } l \in [1, \ldots, N]. \tag{13}$$

At this point, it is worth briefly discussing the physical significance of $\mathcal{Q}$ and $\mathcal{Q}_{\rm E}$. Our framework and in particular our main result – i.e. the equivalence with the maximum entropy ensemble presented in Theorem 3 of the main text – apply for any choice of charges for S and the environment E, given by $\mathcal{Q}$ and $\mathcal{Q}_{\rm E}$ respectively, as long as the total mean value of the compound is preserved. In this sense our results leave open and completely general the choice of conserved quantities. However, one must be cautious by noting that imposing a conservation law of the mean value of $\mathcal{Q} + \mathcal{Q}_{\rm E}$ is not always well-justified. For instance, when $\mathcal{Q}$ are the Hamiltonian, angular momentum and number of particles, it makes sense to allow for environments where $\mathcal{Q}_{{\rm E}^l}$ are the Hamiltonian, angular momentum and number of particles of $\rm E^l$ respectively. In this scenario, imposing Supplementary Equation (12) is meaningful. On the contrary if we take $\mathcal{Q}$ to be the angular momentum and $\mathcal{Q}_{\rm E}$ to be, say, the magnetisation, we find that it might be in general unjustified to impose a conservation of $\mathcal{Q} + \mathcal{Q}_{\rm E}$, since those two quantities are, a priori, unrelated. In summary, our framework takes as a starting point that a conservation law is imposed and builds upon this law. The prior arguments that justify imposing such a conservation law are outside the scope of this paper and must be considered independently.

The definition of $\rho \overset{\boldsymbol{\beta}\text{-mic}}{\rightarrow} \rho_{\rm f}$ is completely analogous to the case of the previous section, with the GGE ensemble playing the role of the canonical ensemble.

*Definition* 2 (Microstate operations with many charges) We say that $\rho_{\rm f}$ can be reached from $\rho_{\rm i}$ by microstate operations, which we denote by

$$\rho_{\rm i} \overset{\boldsymbol{\beta}\text{-mic}}{\rightarrow} \rho_{\rm f}, \tag{14}$$

if for any $\epsilon > 0$ and $\epsilon' > 0$ there exist an environment with observables $\mathcal{Q}_{\rm E}$ and a unitary on SE such that

$$\left\| \left( U(\rho_{\rm i} \otimes \gamma_{\boldsymbol{\beta}}(Q_{\rm E}))U^\dagger \right) - \rho_{\rm f} \right\|_1 \leq \epsilon, \tag{15}$$

while preserving the overall value of the charges

$$\left| \mathrm{tr}\left( U(\rho_{\rm i} \otimes \gamma_{\boldsymbol{\beta}}(Q_{\rm E}))U^\dagger Q^j_{\rm SE} \right) - \mathrm{tr}\left( \rho_{\rm i} \otimes \gamma_{\boldsymbol{\beta}}(Q_{\rm E})\, Q^j_{\rm SE} \right) \right| \leq \epsilon', \tag{16}$$

for all $j = 1, \ldots, n$.

We can now formulate the main result for the case of multiple observables:

*Theorem* 3 (Equivalence with the GGE) Let $\mathcal{Q}$ be any set of commuting observables and the environment be such that $\beta^j \neq 0$ for all $j$. The macrostate $(\mathbf{v}, \mathcal{Q})$ is operationally equivalent to the corresponding GGE ensemble compatible with the partial information $\mathbf{v}$. That is,

$$(\mathbf{v}, \mathcal{Q}) \sim_{\boldsymbol{\beta}} \gamma_{\mathbf{v}}(\mathcal{Q}), \tag{17}$$

where $\mathbf{v}$ are the inverse Lagrange multipliers that one assigns to S so that $\mathrm{tr}(Q^j \gamma_{\mathbf{v}}(\mathcal{Q})) = v^j$ for all $j$.

## Proof of Theorem 3

In this section, we will prove Theorem 3 above, which implies Theorem 3 in the main text as a special case. The equivalence relation in Supplementary Eq. (17) requires showing that

$$(\mathbf{v}, \mathcal{Q}) \overset{\boldsymbol{\beta}\text{-mac}}{\rightarrow} \rho_{\rm f} \Leftrightarrow \gamma_{\mathbf{v}}(\mathcal{Q}) \overset{\boldsymbol{\beta}\text{-mic}}{\rightarrow} \rho_{\rm f}. \tag{18}$$

The direction "⇒" is trivial. Note that the l.h.s. implies that the transition is possible for all initial states compatible with $(\mathbf{v}, \mathcal{Q})$. In particular, $\gamma_{\mathbf{v}}(\mathcal{Q})$ is one of these states compatible with $(\mathbf{v}, \mathcal{Q})$ and hence the r.h.s. condition follows.

Before embarking on the proof of the direction "⇐", we will provide an overview of the different steps involved: 1. We show that macrostate operations allow us to consider without loss of generality probabilistic mixtures of unitary operations as well. 2. We show that using probabilistic mixtures of unitaries, we can reduce the problem to only considering microstates which are diagonal in the basis of the conserved quantities. 3. Using the previous results we show that we can "distill", from the environment described by partial information, systems for which we are certain that they are in the microstates given by the GGE to arbitrary accuracy and with arbitrarily little change of the charges. This shows that we can effectively describe the

environment by GGE microstates directly. 4. We show that once we have an environment directly described by GGE microstates, we can always bring the system to the GGE microstate corresponding to its macrostate. That is, we show that it is possible to implement the transition

$$(\mathbf{v}, \mathcal{Q}) \overset{\boldsymbol{\beta}\text{-mac}}{\to} \gamma_{\mathbf{v}}(\mathcal{Q}). \tag{19}$$

Finally, after we have replaced the state on the system with the GGE by a macrostate operation, we can apply the microstate operation that maps the GGE to the desired final state (r.h.s. of Supplementary Eq. (18) which is the premise of the proof). That is, we compose macrostate operations and microstate operations in the following way:

$$(\mathbf{v}, \mathcal{Q}) \overset{\boldsymbol{\beta}\text{-mac}}{\to} \rho \ \wedge \ \rho \overset{\boldsymbol{\beta}\text{-mic}}{\to} \sigma \Rightarrow (\mathbf{v}, \mathcal{Q}) \overset{\boldsymbol{\beta}\text{-mac}}{\to} \sigma. \tag{20}$$

By taking $\rho = \gamma_{\mathbf{v}}(\mathcal{Q})$ and $\sigma = \rho_{\mathrm{f}}$ and using Supplementary Eq. (19) we obtain the "$\Leftarrow$"-direction, which concludes the proof. We will now give detailed derivations of steps 1.-4. separately.

### Reducing the problem to diagonal microstates

We now show that by being able to implement mixtures of energy-preserving unitaries, we can reduce the problem to one in which all microstates are diagonal in the eigenbasis of all the conserved quantities. To do that, define for every operator $Q^j$ the mixture of unitaries

$$\rho \mapsto \mathcal{D}_{Q^j}(\rho) := \lim_{T\to\infty} \frac{1}{T} \int_0^T \mathrm{e}^{\mathrm{i}Q^j t} \rho \mathrm{e}^{-\mathrm{i}Q^j t} \, \mathrm{d}t. \tag{21}$$

This mixture of unitaries dephases every state in the eigenbasis of $Q_j$. Since all the $Q_j$ commute, we can sequentially apply these maps to map any state $\rho \in [\mathbf{v}]_{\mathcal{Q}}$ to a state that commutes with all $Q^j$. In the following, we will denote this set of microstates that are diagonal in the eigenbasis of all the $Q^j$ and correspond to the macrostate $(\mathbf{v}, \mathcal{Q})$ by $[\mathbf{v}]_{\mathcal{Q}}^{\mathrm{diag}}$. The fact that we can dephase all states without changing the mean values $v^j$ implies that condition Supplementary Eq. (13) of Definition 1 can be relaxed to diagonal states, i.e.,

$$\forall \rho_{\mathrm{i}} \in [\mathbf{v}]_{\mathcal{Q}}^{\mathrm{diag}}, \ \rho_{\mathrm{E}^l} \in [\mathbf{v}_{\boldsymbol{\beta}}(\mathcal{Q}_{\mathrm{E}^l})]_{\mathcal{Q}_{\mathrm{E}^l}}^{\mathrm{diag}} \text{ for } l \in [1, \dots, N]. \tag{22}$$

This allows us to restrict to diagonal states in the last two steps (3. and 4.).

### Mixtures of unitaries

We will now show that instead of considering unitary operations for macrostate operations, for finite temperature environments, we can also use probabilistic mixtures of unitaries. The basic idea is to use systems from the environment, described by the macrostate $\bigotimes_{l=1}^{N} (\mathbf{v}_{\boldsymbol{\beta}}(\mathcal{Q}_{\mathrm{E}^l}), \mathcal{Q}_{\mathrm{E}^l})$, as a source of randomness.

Suppose we want to act with a mixture of unitaries on a system S at hand (which might include other systems from the environment). To do that, we first take two additional systems out of the environment. We choose these subsystems to be qubits labeled by $\mathrm{E}^1$ and $\mathrm{E}^2$ with $\mathcal{Q}_{\mathrm{E}^l} = (H, \mathbb{I}, \dots, \mathbb{I})$. That is, we only consider the energy as a conserved quantity. Let us re-scale their Hamiltonian so that we can write it as $H = 0|0\rangle\langle 0| + \Delta|1\rangle\langle 1|$. As the macrostates have energy $e_\beta(H)$ and they are uncorrelated this determines completely the diagonal of the microstates. One finds that if $\rho_{\mathrm{E}^1} \otimes \rho_{\mathrm{E}^1} \in (e_\beta(H), H)^{\otimes 2}$ then $\mathrm{tr}(\rho_{\mathrm{E}^1} \otimes \rho_{\mathrm{E}^1} |i,j\rangle\langle i,j|) := p_{i,j} = p_i p_j$, with $p_1 = 1 - p_0 = e_\beta(H)/\Delta$. Let us choose $\Delta$ so that $p_0 = 1/\sqrt{2}$.

We now apply to the compound $S\mathrm{E}^1\mathrm{E}^2$ the unitary

$$U = |0,0\rangle\langle 0,0|_{\mathrm{E}^1\mathrm{E}^2} \otimes U_{\mathrm{S}} + (|0,1\rangle\langle 0,1| + |1,0\rangle\langle 1,0| + |1,0\rangle\langle 1,0|)_{\mathrm{E}^1\mathrm{E}^2} \otimes U'_S. \tag{23}$$

One obtains that the effective map on the S is

$$\begin{aligned} \rho \mapsto \mathcal{M}(\rho) &= \mathrm{tr}_{\mathrm{E}^1\mathrm{E}^2}(U\rho U^\dagger) \\ &= p_{0,0} U_{\mathrm{rest}} \rho U_{\mathrm{rest}}^\dagger + (p_{0,1} + p_{1,0} + p_{1,1}) U'_{\mathrm{rest}} \rho U'^\dagger_{\mathrm{rest}} \\ &= (p_0)^2 U_{\mathrm{rest}} \rho U_{\mathrm{rest}}^\dagger + (1 - (p_0)^2) U'_{\mathrm{rest}} \rho U'^\dagger_{\mathrm{rest}} \\ &= \frac{1}{2} U_{\mathrm{rest}} \rho U_{\mathrm{rest}}^\dagger + \frac{1}{2} U'_{\mathrm{rest}} \rho U'^\dagger_{\mathrm{rest}}. \end{aligned} \tag{24}$$

Repeating this process with as many pairs of qubits as required, we can apply apply any mixture of unitaries that we need. Hence, we can assume without loss of generality that in order to perform a macrostate operation as given by Definition 1, it suffices to find, instead of a single unitary $U$ on the SE compound, a mixture of unitaries that performs the desired transition, which we denote as

$$\rho \mapsto \mathcal{U}(\rho) = \sum_{\lambda} p_\lambda U_\lambda \rho U_\lambda^\dagger, \tag{25}$$

with each of $U_\lambda$ preserving the mean value of the conserved quantities.

**From the macrostate environment to the maximum entropy environment**

The macrostate operations and the microstate operations employ different models of the environment. As discussed in the main text, the environment for macrostate operations is given by macrostates of the form

$$\bigotimes_{l=1}^{N} (\mathbf{v}_{\boldsymbol{\beta}}(\mathcal{Q}_{\mathrm{E}^l}), \mathcal{Q}_{\mathrm{E}^l}). \tag{26}$$

On the other hand, for microstate operations one assumes that the environment is given by maximum entropy ensembles of the form

$$\bigotimes_{l=1}^{N'} \gamma_{\boldsymbol{\beta}}(\mathcal{Q}_{\mathrm{E}^l}). \tag{27}$$

We will now show that any environment of the form in Supplementary Eq. (27) can always be "distilled" from an environment of the form in Supplementary Eq. (26). That is, for any $N'$ one can always find a sufficiently large $N$ so that a system of the form in Supplementary Eq. (27) is obtained.

Due to the fact that we can implement mixtures of unitaries and dephase in the energy-eigenbasis we can, without loss of generality, model the macrostate operations that achieve this distillation by mixtures of unitaries that act on diagonal states of the bath, requiring only that they preserve the total expectation values of all the observables. For simplicity, we will take $N' = 1$, since an extension to larger values of $N'$ can be done by simply repeating the process over $N'$ copies of bath macrostates of the form in Supplementary Eq. (26).

For purely technical reasons, we will for now consider the special case where the eigenvalues of all the conserved quantities $Q^j_{\mathrm{E}^l}$ have rational eigenvalues. Since any operator can be approximated to arbitrary accuracy by one with rational eigenvalues, this is not a severe restriction.

Consider a larger number $N$ of identical environment systems in the same macrostate $(\mathbf{v}_\beta(\mathcal{Q}_{\mathrm{E}^l}), \mathcal{Q}^l_{\mathrm{E}})$, where $\mathcal{Q}_{\mathrm{E}^l} = \mathcal{Q}_{E^{l'}}$ for all $l, l' = 1, \ldots, N$. We will apply a unitary map $\mathcal{U}$ of the form in Supplementary Eq. (25) and find that the reduced state on every subsystem is given by $\gamma_{\boldsymbol{\beta}(\mathcal{Q}_{\mathrm{E}^l})}$ to arbitrary accuracy as $N \to \infty$.

We first have to set up some notation. A basis-state on one of the subsystems can be labelled by the eigenvalues $q^j_\alpha$ of the $n$ conserved quantities $Q^j_{\mathrm{E}^l}$, where $\alpha = 1, \ldots, d_{\mathrm{E}^l}(j)$ and $j = 1, \ldots, n$. Here, $d_{\mathrm{E}^l}(j)$ is the number of distinct eigenvalues of $Q^j_{\mathrm{E}^l}$. Simplifying the notation, the basis states on system $E^l$ can thus be labeled by $d$ vectors $\boldsymbol{\alpha}^x = (\alpha^x_1, \ldots, \alpha^x_n)$ corresponding to the choice of eigenvalues $q^j_{\alpha^x_j}$. A basis-state for the $N$ systems is then given by choosing one vector $\boldsymbol{\alpha}^x$ for each subsystem and is denoted by $\boldsymbol{\alpha}^{\mathbf{x}} = (\boldsymbol{\alpha}^{x_1}, \ldots, \boldsymbol{\alpha}^{x_N})$. We will label the joint-eigenspaces of the $Q^j_{\mathrm{E}}$ on the $N$ systems by $\Pi_\xi$ and identify also $\Pi_\xi$ with the projector onto that eigenspace. Given an eigenspace $\Pi_\xi$, we finally denote the corresponding eigenvalue of the total charge $Q^j_{\mathrm{E}}$ as $q^j_{\mathrm{E},\xi}$.

After setting up the notation, we will now start with the actual proof. The operation that we consider is very simple: We simply apply a completely random unitary in each of the subspaces $\Pi_\xi$. This operation clearly commutes with the total charges, hence it also preserves its average value. If we denote the total probability of subspace $\Pi_\xi$ by $p_\xi$, it leaves the whole distribution $p_\xi$ invariant, while leaving each of the subspaces in the maximally mixed state $\Omega_\xi$. Since each of the subspaces is permutation invariant, we find that the state of every system is finally described by the same density matrix

$$\rho'_{\mathrm{E}^l} = \sum_{\xi} p_\xi \, \mathrm{tr}_{\bar{l}}(\Omega_\xi). \tag{28}$$

Since the initial state $\otimes_l \rho_{\mathrm{E}^l}$ is uncorrelated, the total weight of joint eigenspaces $\Pi_\xi$ for which any of the eigenvalues $q^j_{\mathrm{E},\xi}$ deviates by more than $O(\sqrt{N})$ from $N v^j_\beta$ is exponentially small (by Hoeffding's inequality). We will collect the remaining

subspaces in a set $\mathcal{M}$. We thus have

$$\rho'_{\mathrm{E}^l} = \sum_{\xi\in\mathcal{M}} p_\xi \,\mathrm{tr}_{\bar{l}}(\Omega_\xi) + \epsilon_N \sigma, \tag{29}$$

where $\sigma$ is some density-matrix and $\epsilon_N$ goes to zero exponentially with $N$. Note that for all $\xi \in \mathcal{M}$ the corresponding eigenvalues fulfill

$$|q^j_{\mathrm{E},\xi}/N - \mathbf{v}^j_{\boldsymbol{\beta}}| \leq \delta^j_N, \quad \delta^j_N \overset{N\to\infty}{\longrightarrow} 0. \tag{30}$$

We will now show that, as $N \to \infty$, the reduced state on any single subsystem of each of the maximally mixed states $\Omega_\xi$, with $\xi \in \mathcal{M}$, approaches the GGE. To see this pick any such subspace $\Pi_\xi$. The fact that the eigenvalues $q^j_\alpha$ are all rational, together with the fact that $\xi \in \mathcal{M}$ implies that the dimension of any such subspace becomes arbitrarily large with increasing $N$.

Now consider the basis vectors $\boldsymbol{\alpha}^{\mathbf{x}} = (\boldsymbol{\alpha}^{x_1}, \ldots, \boldsymbol{\alpha}^{x_N})$ in $\Pi_\xi$. We will associate to each such basis vector a type

$$T(\boldsymbol{\alpha}^{\mathbf{x}}) = \left(\frac{k_1}{N}, \ldots, \frac{k_d}{N}\right), \tag{31}$$

where $k_x$ is the number of subsystems in state $\boldsymbol{\alpha}^x$. In other words, they fulfill $\sum_x k_x = N$ and

$$\sum_{x=1}^{d_{\mathrm{E}^l}(j)} k_x q^j_{\alpha^x_j} = q^j_{\mathrm{E},\xi}. \tag{32}$$

The number of basis vectors corresponding to the same type $T$ is given by

$$\#T = \frac{N!}{\prod_{x=1}^d k_d}. \tag{33}$$

It can be bounded using Stirling's approximation as

$$\sqrt{2\pi}\mathrm{poly}\,(N)\,\mathrm{e}^{NS(T)} \leq \#T \leq \mathrm{e}\,\mathrm{poly}\,(N)\,\mathrm{e}^{NS(T)},$$

where $S(T) = S(k_1/N, \ldots, k_d/N)$ is the Shannon-entropy of a type. Note that the total dimension of one eigenspace $\Pi_\xi$ is simply given by

$$d(\Pi_\xi) = \sum_{T\in\Pi_\xi} \#T. \tag{34}$$

A type has the property that $T_x = k_x/N \geq 0$ and $\sum_{x=1}^d k_x/N = 1$. It can hence be interpreted as a probability distribution. If the total system is in the state $\Omega_\xi$, we obtain from permutation invariance that the probability to find the $l$-th subsystem in state $\boldsymbol{\alpha}^x$ is given by

$$p^\xi_{\mathrm{E}^l}(\boldsymbol{\alpha}^x) = \frac{\sum_{T\in\Pi_\xi} T_x \#T}{\sum_{T\in\Pi_\xi} \#T}. \tag{35}$$

We will now show that all types that differ from the GGE-distribution by more than $\delta$ (in some norm on $\mathbb{R}^{d-(n+1)}$) have a relative weight that vanishes as $N \to \infty$. In other words, as we increase the system size, the probability distribution $p^\xi_{\mathrm{E}^l}(\boldsymbol{\alpha}^x)$ converges to that of a GGE with $\mathbf{v}^j = q^j_{\mathrm{E},\xi}/N$. Let us denote the probability distribution corresponding to the GGE in subspace $\xi$ by $\gamma_\xi$. Since the Shannon entropy is concave and has a unique maximum among all probability distributions compatible with the expectation values of the conserved quantities $Q^j_{\mathrm{E}^l}$ corresponding to the subspace $\xi$, we can bound the entropy of any type that differs by more than $\delta$ from $\gamma_\xi$ as

$$S(\gamma_\xi) - K'\delta^2 \leq S(T) \leq S(\gamma_\xi) - K\delta^2, \tag{36}$$

where the constants $K$ and $K'$ do not depend on $N$.

We thus see that the weight of the type is

$$\begin{aligned}\sqrt{2\pi}\mathrm{poly}\,(N)\,\mathrm{e}^{NS(\gamma_\xi)-NK'\delta^2} &\leq \#T \\ &\leq \mathrm{e}\,\mathrm{poly}\,(N)\,\mathrm{e}^{NS(\gamma_\xi)-NK\delta^2}.\end{aligned}$$

Hence, the weight of the types is distributed according to a Gaussian-distribution on a subset of $\mathbb{R}^{d-(n+1)}$ with variance $\sigma^2$ of order $1/N$. For large $N$, it is thus very sharply peaked around the Gibbs-distribution and we can choose $\delta$ to go to 0 as $N \to \infty$ while at the same time most of the weight of the distribution is carried by distribution within $\delta$ away from the GGE distribution. Choose, for example, $\delta = N^{1/4}\sigma$, so that

$$\lim_{N\to\infty} N^{1/4}\sigma = \lim_{N\to\infty} N^{1/4-1/2} = \lim_{n\to\infty} N^{-1/4} = 0. \tag{37}$$

More formally, we can upper bound the total weight of types more than $\delta$ away from the GGE distribution by

$$\sum_{\substack{T\in\Pi_\xi,\\ \|T-\gamma_\xi\|_1\geq\delta}} \#T \leq \mathcal{T}_\xi \, e \, \mathrm{poly}\,(N)\, \mathrm{e}^{NS(\gamma_\xi)-NK\delta^2}, \tag{38}$$

where $\mathcal{T}_\xi$ is the total number of different types appearing in subspace $\Pi_\xi$. Similarly, for any $q < 1$ we can lower bound the total weight of types closer than $q\delta$ to the GGE distribution by

$$\sum_{\substack{T\in\Pi_\xi,\\ \|T-\gamma_\xi\|_1\leq q\delta}} \#T \geq \mathrm{poly}\,(q\delta)\, \mathcal{T}_\xi \sqrt{2\pi}\, \mathrm{poly}\,(n)\, \mathrm{e}^{NS(\gamma_\xi)-NK'q^2\delta^2}.$$

The relative volume of the two is then given by (using $\delta = N^{-1/4}$)

$$\begin{aligned}\frac{\mathrm{e}\,\mathrm{poly}\,(N)\,\mathrm{e}^{NS(\gamma_\xi)-NK\delta^2}}{\sqrt{2\pi}\mathrm{poly}\,(q\delta)\,\mathrm{poly}\,(N)\,\mathrm{e}^{NS(\gamma_\xi)-NK'\delta^2q^2}} &= \frac{\mathrm{e}\,\mathrm{poly}\,(N)\,\mathrm{e}^{NS(\gamma_\xi)-\sqrt{N}K}}{\sqrt{2\pi}\mathrm{poly}\,\left(qN^{-1/4}\right)\mathrm{poly}\,(N)\,\mathrm{e}^{NS(\gamma_\xi)-\sqrt{N}K'q^2}} \\ &\leq K''\mathrm{poly}\,(N)\,\mathrm{e}^{-\sqrt{N}(K-K'q^2)} \to 0,\end{aligned} \tag{39}$$

for $q < \sqrt{K/K'}$. As $N \to \infty$, we therefore find that

$$\begin{aligned}\lim_{N\to\infty} \mathrm{tr}_{\bar{l}}\,(\Omega_\xi) &= \lim_{n\to\infty} \sum_x p^\xi_{\mathrm{E}^l}(\boldsymbol{\alpha}^x)|\boldsymbol{\alpha}^x\rangle\langle\boldsymbol{\alpha}^x| \\ &= \lim_{N\to\infty} \gamma_{\boldsymbol{\beta}_\xi}(\mathcal{Q}_{\mathrm{E}^l}) \\ &= \gamma_{\boldsymbol{\beta}}(\mathcal{Q}_{\mathrm{E}^l}),\end{aligned} \tag{40}$$

where $\boldsymbol{\beta}_\xi$ is the vector of "inverse temperatures" corresponding to the subspace $\Pi_\xi$ and in the last line we have used that $\lim_N q^j_{\mathrm{E},\xi}/N = \mathbf{v}^j_{\boldsymbol{\beta}}$ for all $\xi \in \mathcal{M}$. Since this holds for all subspaces in $\mathcal{M}$, we finally obtain the desired result that

$$\rho'_{\mathrm{E}^l} = \sum_{\xi\in\mathcal{M}} p_\xi \,\mathrm{tr}_{\bar{l}}(\Omega_\xi) + \epsilon_N\sigma \overset{N\to\infty}{\longrightarrow} \gamma_{\boldsymbol{\beta}}(\mathcal{Q}_{\mathrm{E}^l}). \tag{41}$$

Concluding, we have shown that by taking many copies of the macrostate $(\mathbf{v}_{\boldsymbol{\beta}}, \mathcal{Q})$ and applying an exactly energy-conserving operation, we can prepare the microstate $\gamma_{\boldsymbol{\beta}}(\mathcal{Q})$. Repeating this process many times, we can then also prepare any environment of the form

$$\bigotimes_l \gamma_{\boldsymbol{\beta}}(\mathcal{Q}_{\mathrm{E}^l}). \tag{42}$$

**Bringing the system to the maximum entropy state using the maximum entropy environment**

In the last section we have proven that, from the model of the environment given by the form in Supplementary Eq. (26) for the definition of macrostate operations, one can distill a microstate environment of the form in Supplementary Eq. (27). We will now use such an environment to bring the system to the maximum entropy state. That is, to perform the transition in Supplementary Eq. (19). The idea to do that is very simple: We choose the right conserved quantities $Q_{\mathrm{E}}$ on the environment and then simply swap the system state with the environment.

Suppose that the system is in macrostate $(\mathbf{v}, \mathcal{Q})$ with conserved quantities $Q^j$ and let the corresponding inverse temperatures given by $\gamma_{\mathbf{v}}(\mathcal{Q})$ be given by $\beta_j(\mathbf{v})$. Now choose the following conserved quantities on the environment,

$$Q^j_{\mathrm{E}} = \frac{\beta_j(\mathbf{v})}{\beta_j} Q^j. \tag{43}$$

Of course, this is possible only if $\beta_j \neq 0$ for all $j$. Then the two density matrices of the GGEs coincide, $\gamma_{\boldsymbol{\beta}}(\mathcal{Q}_{\rm E}) = \gamma_{\boldsymbol{\beta}(\mathbf{v})}(\mathcal{Q})$, and hence the total charge is conserved on average as the two states are swapped (it is not conserved exactly, since the microstate on the system can be any microstate in $[\mathbf{v}]_{\mathcal{Q}}$). As mentioned in the previous section, the above reasoning strictly speaking only applies if the eigenvalues of $Q^j_{\rm E}$ are rational. However, we can always approximate $Q^j_{\rm E}$ by an operator with rational eigenvalues to arbitrary precision. In this case, the average charge conservation is fulfilled with arbitrary precision as well.

### Non-Gibbsian average energies trivialize thermodynamics

In this section, we will show that the assignment of macrostates to the environment as in Eq. $(4)$ of the main text is the only one that does not lead to i) arbitrary work extraction from the environment and ii) trivial macrostate operations, in the sense that any transition is possible. For this, we will analyse the consequences of having an assignment of energies given by $\mathrm{f}(H)$ different from the one we assume for $e_\beta(H)$. For simplicity we will discuss it for the case of the energy as a single conserved quantity, since the argument is fully analogous for the case of other conserved quantities.

Let us first show i). The function $f$ can, without loss of generality, be always expressed as $f(H) = e_{\beta(H)}(H)$, where now $\beta(H)$ is not a fixed value but a function of the Hamiltonian. For the situation to not be equivalent to some fixed inverse temperature, at least two Hamiltonians must have different temperatures, i.e., there exist Hamiltonians $H_1 \neq H_2$ such that $\beta(H_1) \neq \beta(H_2)$. For simplicity let us write $\beta_j = \beta(H_j)$ in the following. Given any value of $\beta_j$ we can distill, from a large number of macrostates of the environment, one canonical ensemble at temperature $\beta_j$. That is, from an environment of the form

$$\bigotimes_{j=1}^{N_1}(e_{\beta_1}(H_1), H_1)\bigotimes_{j=1}^{N_2}(e_{\beta_2}(H_2), H_2) \tag{44}$$

one can obtain systems in the microstate

$$\gamma_{\beta_1}(H_1)^{\otimes N_1'} \otimes \gamma_{\beta_2}(H_2)^{\otimes N_2'} \tag{45}$$

with $N_1'$ and $N_2'$ arbitrarily large for sufficiently large $N_1$ and $N_2$. Once we possess two systems in the canonical ensemble at different inverse temperatures $\beta_1$ and $\beta_2$, one can trivially extract work. That is, one could reduce the mean energy of Supplementary Eq. (45) and accumulate it in a work storage device. This is true since for some value for $N_1'$ and $N_2'$, Supplementary Eq. (45) will cease to be a passive state [1].

The previous considerations imply trivially ii). Once we have established that the environment could be used to extract an arbitrary amount of work –mean energy–, one can invest this energy in creating an arbitrary state [2]. Hence one finds that if $f(H)$ is not the thermal energy, then

$$(e, H) \overset{\beta\text{-mac}}{\to} \rho. \tag{46}$$

is possible for any $\rho$.

Altogether, we conclude that imposing that i) or ii) are impossible implies that $f(H) = e_\beta(H)$ for a fixed $\beta$. In other words, there only exist specific families of functions, one for each value of $\beta$, that do not lead to trivial macrostate operations or work extraction from the environment. In this way the assignment of a parameter $\beta$ to the environment follows from those basic principles. Importantly, note that the parameter $\beta$ is in principle not related to any prior assignment of a temperature to the environment. For the sake of simplicity, we refer to $\beta$ as the inverse temperature, but the interpretation of $\beta$ as related to a prior value of $T$ as $\beta = (\mathrm{k_B}T)^{-1}$ is not necessary to derive Theorem 3 of the Supplementary Material or any of the results in this work. In summary, we conclude that the only thermodynamically consistent way to assign average energies to environment systems is by assigning the energies corresponding to a thermal Gibbs state for some parameter $\beta$ playing the role of an inverse temperature.

## SUPPLEMENTARY METHODS 2
## BREAKDOWN OF EQUIVALENCE UNDER EXACT ENERGY CONSERVATION

In this section, we will prove the inequivalence between macrostates and their corresponding maximum-entropy ensemble when exact energy conservation, (28) in the main text, is imposed. In particular, we show that for every $\beta$ and non-trivial $H$, there exists at least one initial value $e$, such that

$$(e, H) \overset{\mathrm{c}}{\not\approx}_\beta \gamma_e(H). \tag{47}$$

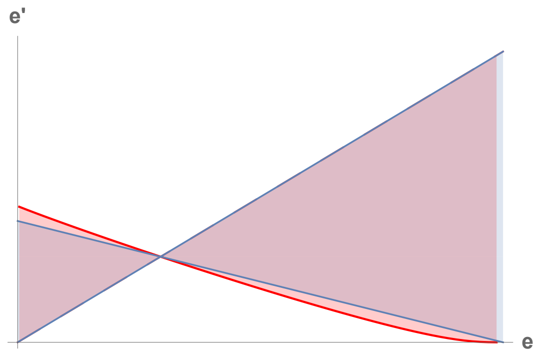


**Figure 1:** Set of reachable final energies $e'$, given some Hamiltonian $H$ and initial energy $e$. The reachable final energies under commuting macrostate operations are upper bounded by two lines (blue region) that themselves lower bound the set of reachable energies under microstate commuting operations (red region). The results of Ref. [4] imply that, for any non-trivial $H$ and $\beta$, the red region has a non-linear boundary, which further implies that the blue region is strictly smaller than the red region. This, in turn, immediately gives Supplementary Eq. (52) and, hence, yields the breakdown of operational equivalence. In this figure, the intersection point marks the thermal energy $e_\beta(H)$, that is a fixed point of all operations by definition. Note further that the two sets are bounded, in one direction, by the identity. This follows from free energy considerations.

Let us first introduce some notation. We define commuting macrostate operations, denoted by

$$(e, H) \overset{\beta\text{-c-mac}}{\to} \rho_{\mathrm{f}}, \tag{48}$$

similarly to Definition 1 of the main text but replacing condition (9) in the main text by $[U, H_{\mathrm{SE}}] = 0$. In a similar fashion, we define commuting microstate operations, denoted by

$$\rho \overset{\beta\text{-c-mic}}{\to} \rho_{\mathrm{f}}, \tag{49}$$

similarly to Definition 2 in the main text but replacing condition (12) in the main text by $[U, H_{\mathrm{SE}}] = 0$. Commuting microstate operations are in the literature discussed as "thermal operations" [3, 4]. Proving the inequivalence in Supplementary Eq. (47) amounts to finding one microstate $\sigma$ so that

$$(e, H) \overset{\beta\text{-c-mac}}{\not\to} \sigma, \tag{50}$$

$$\gamma_e(H) \overset{\beta\text{-c-mic}}{\to} \sigma. \tag{51}$$

The existence of such a state $\sigma$ is implied by the fact that, for any non-trivial $H \neq 0$ and any $\beta$, there exists at least one initial energy $e$ such that

$$\max_{(e,H)\overset{\beta\text{-c-mac}}{\to}\rho_{\mathrm{f}}} \mathcal{E}(\rho_{\mathrm{f}}) < \max_{\gamma_e(H)\overset{\beta\text{-c-mac}}{\to}\rho_{\mathrm{f}}} \mathcal{E}(\rho_{\mathrm{f}}). \tag{52}$$

This equation implies the existence of $\sigma$ because, if $\sigma$ did not exist, then the reachable energies under the two types of operations would coincide. The equation itself follows from a result that we present in the next section and in which the reachable energies under macrostate commuting operations are linearly upper bounded, as illustrated in Fig. 1. We believe that this bound may be of independent interest.

### Partial characterisation of commuting macrostate transitions

In this section we will provide a method to analyse the allowed transitions under commuting macrostate operations. We cannot in general provide a full answer to which transitions $(e, H) \overset{\beta\text{-c-mac}}{\to} \rho_{\mathrm{f}}$ are possible. However, we will provide a method to bound the maximum and minimum energies of the states $\rho_{\mathrm{f}}$ achievable from a given macrostate $(e, H)$.

First, we need to consider a set of transitions between macrostates that are closely related to those produced by commuting macrostate operations:

*Definition* 4 (Macrostate GP-maps) We say that $(e', H)$ can be reached from $(e, H)$ by macrostate GP-maps, which we denote by $(e, H) \overset{\beta\text{-mGP}}{\to} (e', H)$, if for any $\epsilon > 0$ there exists a completely positive, trace preserving (CPTP)-map $G$ such that

1. $G(\gamma_\beta(H)) = \gamma_\beta(H)$,
2. $G(\rho) \in [e']^\epsilon_H, \quad \forall \rho \in [e]_H$ .

Here, $[e']^\epsilon_H$ denotes the union of the equivalence classes that differ from $e'$ by at most $\epsilon$. By definition of the operations, and from results in Ref. [5], the following chain of implications holds: For any $\rho \in [e']_H$,

$$(e, H) \overset{\beta\text{-c-mac}}{\to} \rho \quad \Rightarrow (e, H) \overset{\beta\text{-mGP}}{\to} (e', H), \tag{53}$$

$$\Rightarrow \gamma_e(H) \overset{\beta\text{-c-mic}}{\to} \gamma_{e'}(H). \tag{54}$$

This in turn implies that for all $(e, H)$,

$$\max_{(e,H)\overset{\beta\text{-c-mac}}{\to}\rho_{\rm f}} \mathcal{E}(\rho_{\rm f}) \leq \max_{(e,H)\overset{\beta\text{-mGP}}{\to}(e',H)} e' \leq \max_{\gamma_e(H)\overset{\beta\text{-c-mic}}{\to}\rho_{\rm f}} \mathcal{E}(\rho_{\rm f}). \tag{55}$$

From the results of Ref. [4] it follows that the rightmost term in the last equation is a non-linear function of $e$. In contrast, for the middle term, we find the following lemma.

*Lemma* 5 (Reachable energies under macrostate GP-maps) For any non-trivial $H$ and $\beta$, if $e \in ]e_{\min}, e_{\max}[$,

$$\max_{(e,H)\overset{\beta\text{-mGP}}{\to}(e',H)} e' = \begin{cases} e & \text{if } e \geq e_\beta(H), \\ e_\beta(H) + \alpha(e) K_{\beta,H} & \text{if } e < e_\beta(H), \end{cases} \tag{56}$$

where $e \mapsto \alpha(e)$ is a function linear in $e$ and $K_{\beta,H}$ is a constant independent of $e$. Similarly,

$$\min_{(e,H)\overset{\beta\text{-mGP}}{\to}(e',H)} e' = \begin{cases} e_\beta(H) + \alpha(e) K_{\beta,H} & \text{if } e \geq e_\beta(H), \\ e & \text{if } e < e_\beta(H). \end{cases} \tag{57}$$

This lemma characterizes the set of reachable energies under macrostate GP-maps, and hence upper and lower bounds the possible state transitions under commuting macrostate and microstate operations respectively. As discussed below, the constant $K_{\beta,H}$ can easily be evaluated as a linear program. With respect to Supplementary Eq. (55), Lemma 5 and the results from Ref. [4] together imply that the second inequality in the equation has to be strict for at least one initial energy $e \in ]e_{\min}, e_{\max}[$ and hence that Supplementary Eq. (52) holds.

**Proof of Lemma 5**

Denote the set of macrostate GP-maps for a given initial energy $e$ as $\mathcal{G}_e$. First, note that just like in the previous proofs, we need to consider only microstates $\rho \in [e]^{\rm diag}_H$ that are diagonal in the eigenbasis of $H$, because the decoherence map $\mathcal{U}_{dec.}$ defined in Supplementary Eq. (21) is clearly a macrostate GP-map (mapping a macrostate to itself). Next, let

$$\mathcal{N} = \{A | \text{diag}(A) = A \wedge \text{tr}(H^\dagger A) = 0 \wedge \text{tr}(A) = 0\} \tag{58}$$

be the space of traceless, diagonal matrices that are orthogonal to $H$, for which $\dim(\mathcal{N}) = d - 2$. Further, let $T$ be the matrix that is orthogonal to both $H$ and $\mathcal{N}$ and for which $\text{tr}(H) = \text{tr}(T)$. This matrix always exists. Clearly, if $\{N_i\}_{i=1}^{d-2}$ is some orthogonal basis of $\mathcal{N}$, then $\{H, T, N_1, \ldots, N_{d-2}\}$ form a complete basis of the diagonal sector. For this reason, we can expand any diagonal state $\rho$ as

$$\rho = \gamma_e(H) + \alpha(e)(H - T) + N(\rho), \tag{59}$$

where

$$\alpha(e) = \frac{e - e_\beta(H)}{\text{tr}(H^2)}, \tag{60}$$

$N(\rho) \in \mathcal{N}$. Furthermore, by construction, in this expansion, any two states from the same equivalence class differ only by an element in $\mathcal{N}$. This expansion is useful because it allows us to show the following lemma.

*Lemma* 6 (Characterising initial states in macrostate GP-maps) For non-trivial $H$ and for any $e \in ]e_{\min}, e_{\max}[$, a CPTP-map satisfies condition 2 from Definition 4 iff $G(\mathcal{N}) \subseteq \mathcal{N}$.

*Proof.* $\Leftarrow$: Suppose there exists a map $G$ and some state $\rho \in [e]_H^{\text{diag}}$ such that

$$G(\rho) \in [e']_H. \tag{61}$$

If $G[\mathcal{N}] \subseteq \mathcal{N}$, then for any other state $\rho' \in [e]_H^{\text{diag}}$,

$$\begin{aligned}\mathcal{E}(G(\rho')) &= \mathcal{E}(G(\rho)) + \mathcal{E}(G(N)) \\ &= e' + \mathcal{E}(N) \\ &= e',\end{aligned} \tag{62}$$

and hence $G$ satisfies condition 2.

$\Rightarrow$: Suppose that $G \in \mathcal{G}_e$. Then, for any $\rho, \rho' \in [e]_H^{\text{diag}}$, by Supplementary Eq. (59)

$$\rho - \rho' = N, \tag{63}$$

$$G(\rho) - G(\rho') = N', \tag{64}$$

and hence, by the linearity of CPTP-maps

$$\begin{aligned}G(N) &= G(\rho - \rho') \\ &= G(\rho) - G(\rho') \\ &= N'.\end{aligned} \tag{65}$$

This implies that $G[\mathcal{N}_e] \subseteq \mathcal{N}$, where

$$\mathcal{N}_e = \{N \in \mathcal{N} | \exists \rho, \rho' \in [e]_H^{\text{diag}} : \rho + N = \rho'\}. \tag{66}$$

$\mathcal{N}_e$ is the subspace of elements in $\mathcal{N}$ that connect elements from $[e]_H^{\text{diag}}$ with another. Now, for any $e \in ]e_{\min}, e_{\max}[$, that is, any non-extremal initial energy, it follows from the simplex geometry of the space of diagonal states that $\dim(\mathcal{N}_e) = \dim([e]_H^{\text{diag}}) = \dim(\mathcal{N})$. But this implies that there exists a complete ($(d-2)$-dimensional basis) $\{N_i\}$ of $\mathcal{N}_e$ that also constitutes a basis for $\mathcal{N}$. Hence, $G[\mathcal{N}_e] \subseteq \mathcal{N}$ implies $G[\mathcal{N}] \subseteq \mathcal{N}$.

□

Note that for $e \in \{e_{\min}, e_{\max}\}$, depending on the degeneracy of the Hamiltonian $H$, it may be the case that $\dim(\mathcal{N}_e) = \dim([e]_H^{\text{diag}}) = 0 \neq \dim(\mathcal{N})$, so that the above lemma is not guaranteed to hold for extremal energies. Lastly, note that by the same reasoning, the proof holds also if we consider restricted sets of states strictly contained in $[e]_H^{\text{diag}}$, as long as the restricted set spans a vector space of the same dimensionality as the one spanned by $[e]_H^{\text{diag}}$. This has as a consequence that the breakdown of equivalence as phrased in the previous section as well as the results in this section hold if one further restricts the set of possible states in the equivalence class to an $\epsilon$-ball around a given state, which also spans a vector space of the right dimensionality.

To proceed, a corollary of Lemma 6 is that the set of macrostate GP-maps is the same, regardless of the initial energy, i.e. $\mathcal{G}_e = \mathcal{G}_{e'}$, for any $e, e' ]e_{\min}, e_{\max}[$. This allows us to drop the index in the following. Then, by Supplementary Eq. (59) we have

$$\begin{aligned}\max_{(e,H) \overset{\beta\text{-mGP}}{\to} (e',H)} e' &= \max_{G \in \mathcal{G}} \mathcal{E}(G(\rho)), \rho \in [e]_H^{\text{diag}} \\ &= \max_{G \in \mathcal{G}} \mathcal{E}(\gamma_e(H) + \alpha(e) G(H-T) + G(N(\rho))) \\ &= e_\beta(H) + \max_{G \in \mathcal{G}} \alpha(e) \mathcal{E}(G(H-T)).\end{aligned} \tag{67}$$

Finally, note that

$$\max_{G \in \mathcal{G}} \alpha(e) \mathcal{E}(G(H-T)) = \begin{cases} \alpha(e) \max_{g \in \mathcal{G}} \mathcal{E}(G(H-T)), & \text{if } e \geq e_\beta(H), \\ \alpha(e) \min_{g \in \mathcal{G}} \mathcal{E}(G(H-T)), & \text{if } e < e_\beta(H), \end{cases} \tag{68}$$

because $\alpha(e)$ flips sign around $e_\beta(H)$. Defining the constants

$$F_{\beta,H} = \max_{g\in\mathcal{G}} \mathcal{E}(G(H-T)), \tag{69}$$

$$K_{\beta,H} = \min_{g\in\mathcal{G}} \mathcal{E}(G(H-T)), \tag{70}$$

we then have

$$\max_{(e,H)\overset{\beta\text{-mGP}}{\to}(e',H)} e' = \begin{cases} e_\beta(H) + \alpha(e)F_{\beta,H}, & \text{if } e \geq e_\beta(H), \\ e_\beta(H) + \alpha(e)K_{\beta,H}, & \text{if } e < e_\beta(H). \end{cases} \tag{71}$$

Similarly,

$$\min_{(e,H)\overset{\beta\text{-mGP}}{\to}(e',H)} e' = \begin{cases} e_\beta(H) + \alpha(e)K_{\beta,H}, \text{ if } e \geq e_\beta(H), \\ e_\beta(H) + \alpha(e)F_{\beta,H}, \text{ if } e < e_\beta(H). \end{cases} \tag{72}$$

In the final step, we will now discuss the values of $F_{\beta,H}$ and $K_{\beta,H}$. The former can be found analytically to be such that

$$e_\beta(H) + \alpha(e)F_{\beta,H} = e. \tag{73}$$

To see this, note that the upper term in Supplementary Eq. (71) denotes the maximum reachable energy if the initial energy lies above the thermal energy (see Fig. 1). This is trivially is at least $e$ (because the identity is always a macrostate GP-map). Now, if it was the case that

$$e_\beta(H) + \alpha(e)F_{\beta,H} > e, \tag{74}$$

then this would imply that there exists a GP-map $G$ such that

$$\mathcal{E}(G(\gamma_e(H))) > e. \tag{75}$$

In this case, $G$ would have certainly increased the free energy $\Delta F(\rho) := S(\rho||\gamma_\beta(H))$ of the system, by monotonicity of the free energy of thermal states in $e$: For any $e' > e, \rho \in [e']_H$,

$$\Delta F(\gamma_e(H)) < \Delta F(\gamma_{\beta_S(e')}(H)) \leq \Delta F(\rho). \tag{76}$$

Results from Ref. [5] imply that no GP-map can increase the free energy of the system, so that Supplementary Eq. (74) cannot be true, and hence $F_{\beta,H}$ is determined by Supplementary Eq. (73).

Regarding $K_{\beta,H}$, it cannot in general be fixed analytically and depends on $H$ and $\beta$. However, it can readily be computed with a linear program. This is because for any initial energy $e$, the optimization problems stated in Supplementary Equations (71) and (72) can be cast as linear programs. This is true since achievable state transitions under general GP-maps can be formulated as an LP [5, 6], and Lemma 6 shows that the only further constraint on macrostate GP-maps is itself linear, namely that $\mathcal{G}(\mathcal{N}) \subseteq \mathcal{N}$. Finally, note also that a similar Lemma to Lemma 6 can be shown to hold true for several commuting observables $\mathcal{Q}$. There, each of the observables $Q^j$ is bounded linearly, so that, in total, the reachable states will be characterized by piece-wise linear bounds, instead of a single linear bound. Since this lemma is a straightforward generalization of Lemma 5, we omit its proof here.

**Local asymptotic equivalence in the commuting case**

In this section we show that, locally and asymptotically, one can recover operational equivalence for the scenario in which both macrostate and microstate operations are commuting. Consider an $N$-partite, non-interacting system with initial macrostate $\bigotimes_{l=1}^N (e,H)$, that is, all parts share the same local Hamiltonian and initial energy. We will now use the results of the Supplementary Methods 1. There, it is shown that by means of commuting macrostate operation, one can bring a bath given by the macrostate of Supplementary Eq. (26) with $N \to \infty$ to a final state $\rho_f$ such that all its reduced states are arbitrarily close in trace norm to the maximum-entropy ensemble compatible with the partial information (see Supplementary Eq. (41)). We can now apply the same operation to the system in macrostate $\bigotimes_{l=1}^N (e,H)$. Formally,

$$\bigotimes_{l=1}^{N} (e,H) \overset{\beta\text{-c-mac}}{\to} \rho_{\mathrm{f}}, \tag{77}$$

such that

$$\mathrm{tr}_{\bar{l}}(\rho_{\mathrm{f}}) \stackrel{N\to\infty}{\longrightarrow} \gamma_e(H). \tag{78}$$

Note that the state $\rho_{\mathrm{f}}$ will be very different from the global canonical ensemble state $\bigotimes_l \gamma_e(H)$ (that is reachable from the usual macrostate operations but not with commuting macrostate operations), since the different sites will in general be highly correlated.

Nevertheless, in direct analogy to the reasoning in the case of average-energy preserving operations, we can now apply any set of commuting microstate operations that act on the individual sites $l$, to prepare, locally, any state that could have been reached if instead one had started with the canonical ensemble states $\gamma_e(H)$ on $l$. In this sense, local operational equivalence for the commuting case is recovered for i.i.d. and non-interacting systems in the thermodynamic limit. Note also that these results do not change if we additionally allowed access to Gibbs states on the bath, instead of macrostates, since we can again use the distillation procedure that we used before to arbitrarily well prepare Gibbs states $\gamma$ using commuting operations. Also, as with the other results, this argument extends to the case of several commuting observables.

## SUPPLEMENTARY METHODS 3
## MACROSTATE OPERATIONS IN THE MACROSCOPIC LIMIT

In this section we discuss the value of the higher moments of the energy difference $X$ when performing a macrostate operation. As stated in the main text, we assume that $H = \sum_i H^i$. We first consider the case of a system whose subsystems are uncorrelated. That is, we assume the initial system macrostate to be of the form $(e, H) = \otimes_{i=1}^N (e_i, H^i)$. The canonical ensemble state for $(e, H)$ is

$$\gamma_e(H) = \bigotimes_{i=1}^N \gamma_{\frac{e}{N}}(H^i). \tag{79}$$

Finally, we consider a macrostate transition $(e, H) \stackrel{\beta-\mathrm{mac}}{\rightarrow} \rho_{\mathrm{f}}$, where we also assume that

$$\rho_{\mathrm{f}} = \bigotimes_{i=1}^N \rho_{\mathrm{f}}^i. \tag{80}$$

We are interested in the distribution $P(X)$, where $X$ is the change in energy under this macrostate transition.

To see that $P$ will be normally distributed, we implement the above transition by acting on each of the subsystems independently. By Theorem 3 of the main text, we know that this is possible. In particular, by the procedure presented in the Supplementary Methods 1, we can implement the transition

$$(e_i, H^i) \stackrel{\beta-\mathrm{mac}}{\rightarrow} \gamma_e(H^i) \tag{81}$$

as a macrostate transition, for any subsystem $i$. This produces a change in energy $X_i$ with mean $\mu_i$ and variance $\sigma_i^2$, which is finite for bounded $H_i$. Let $s_N^2 = \sum_i^N \sigma_i^2$. Then, by the Lyapunov Central Limit Theorem, we have that the total change in energy, $X = \sum_i X_i$, converges in distribution to a normal distribution,

$$\lim_{N\to\infty} X \stackrel{d}{\rightarrow} \mathcal{N}(\sum_i \mu_i = e' - e, s_N^2), \tag{82}$$

with $e'$ being the final energy of the system, if the following condition is satisfied: There exists a $\delta > 0$ such that

$$\lim_{N\to\infty} \frac{1}{s_N^{2+\delta}} \sum_i^N \mathbb{E}[|X_i - \mu_i|^{2+\delta}] = 0. \tag{83}$$

Choosing $\delta = 1$ and since $s_N^2 = O(N)$, this is satisfied if $\sum_i^N \mathbb{E}[|X_i - \mu_i|^{2+\delta}] = O(N)$. This is a physically reasonable assumption to make. Now, from Supplementary Eq. (82) it follows that the energy change per subsystem is normally distributed as

$$\lim_{N\to\infty} \frac{X}{N} \stackrel{d}{\rightarrow} \mathcal{N}(e' - e, \frac{s_N^2}{N}). \tag{84}$$

In terms of the higher moments this means the following. Let

$$\mu_n(X) := \mathbb{E}[(X-\mu)^n], \quad n \in 1, 2, \dots \tag{85}$$

be the moments of a random variable $X$. If this $X$ is normally distributed with variance $\sigma^2$, then independent of its mean the following is true and can be verified by evaluation.

$$\mu_{2n}(X) = \sigma^{2n}(2n-1)!!, \quad \mu_{2n+1}(Y) = 0. \tag{86}$$

Combining this with Supplementary Eq. (84) we find that the higher moments per subsystem vanish in the macroscopic limit:

$$\lim_{N\to\infty} \mu_{2n}(X/N) = \lim_{N\to\infty} \left(\frac{s_N}{\sqrt{N}}\right)^{2n} (2n-1)!! = 0. \tag{87}$$

As stated in the main text, this can be seen as an argument in favour of the assignment of the ensemble to macrostates, for large weakly-correlated systems, as long as one tolerates violations of $(28)$ in the main text – as measured by the higher moments – that are negligible in comparison with the typical energy scales involved in the thermodynamic operation. Of course, a similar argument can be made for the case of weakly correlated systems. However, for conceptual clarity we here restricted to the independent case.

## REFERENCES

[1] Pusz, W. & Woronowicz, S. L. Passive states and KMS states for general quantum systems. Commun. Math. Phys. **58**, 273–290 (1978).
[2] Skrzypczyk, P., Short, A. J. & Popescu, S. Work extraction and thermodynamics for individual quantum systems. Nature Comm. **5**, 4185 (2016).
[3] Brandão, F. G. S. L., Horodecki, M., Ng, N. H. Y., Oppenheim, J. & Wehner, S. The second laws of quantum thermodynamics. PNAS **112**, 3275–3279 (2015).
[4] Horodecki, M. & Oppenheim, J. Fundamental limitations for quantum and nanoscale thermodynamics. Nature Comm. **4**, 2059 (2013).
[5] Janzing, D., Wocjan, P., Zeier, R., Geiss, R. & Beth, T. Thermodynamic cost of reliability and low temperatures: Tightening Landauer's principle and the second law. Int. J. Th. Phys. **39**, 2717–2753 (2000).
[6] Renes, J. M. Work cost of thermal operations in quantum and nano thermodynamics. Eur. J. Phys. Plus **129**, 153 (2014).
